# Influence of the Type of Soft Segment on the Selected Properties of Polyurethane Materials for Biomedical Applications

**DOI:** 10.3390/ma17040840

**Published:** 2024-02-09

**Authors:** Andrzej Puszka, Janusz W. Sikora, Aleksandra Nurzyńska

**Affiliations:** 1 Department of Polymer Chemistry, Institute of Chemical Sciences, Faculty of Chemistry, Maria Curie-Skłodowska University in Lublin, Gliniana Street 33, 20-614 Lublin, Poland; 2 Department of Technology and Polymer Processing, Faculty of Mechanical Engineering, Lublin University of Technology, Nadbystrzycka Street 36, 20-618 Lublin, Poland; janusz.sikora@pollub.pl; 3 Chair and Department of Biochemistry and Biotechnology, Faculty of Pharmacy, Medical University of Lublin, Chodzki Street 1, 20-093 Lublin, Poland; aleksandra.nurzynska@umlub.pl

**Keywords:** polyurethane, antimicrobial activity, cytotoxicity, TGA-FTIR

## Abstract

This work presents the synthesis and characterization of new TPUs obtained by melt polyaddition using 1,1′-methanediylbis(4-isocyanatocyclohexane) (HMDI, *Desmodur W*^®^), a new unconventional chain extender, i.e., (methanediyldibenze-ne-4,1-diyl)dimethanediol, and five types of soft segments differing in structure and molar masses. The structure of the obtained polymers was determined (by using the Fourier transform infrared spectroscopy and X-ray diffraction methods), and the physicochemical (reduced viscosity, density), optical (UV-VIS), processing (MFR) and thermal (DSC and TGA-FTIR) as well as surface, antibacterial and cytotoxic properties were determined. Based on the results obtained, it can be stated that the type of soft segment used significantly affects the properties of the obtained polymers. The most favorable properties for use in medicine were demonstrated by materials based on a polycarbonate soft segment.

## 1. Introduction

Polyurethanes (PURs) are the most multipurpose family of polymeric materials due to the fact that they are used in many forms and have many end uses. They are generally produced in the form of coatings, elastomers, adhesives, foams, fibers, synthetic leathers and others and are used in the construction and automotive industries and footwear production as well as medical technology [1,2,3]. In recent years, the polyurethane industry has experienced significant development, with particularly rapid development in the area of elastomers. This increase in demand for elastomers is due to the unique set of properties of this class of PUR, such as good abrasion, impact and chemical resistance, as well as high tensile and tear strength. All these special qualities and the processability of thermoplastic materials are joined in thermoplastic polyurethane elastomers (TPUs), an extremely interesting group of polyurethane materials. TPUs are multi-block copolymers with an alternating sequence of hard and soft segments [2,3,4,5]. In typical TPUs, hard segments are made as a result of the reaction of short-chain aliphatic diols (referred to as chain extenders), mainly butane-1,4-diol, with diisocyanates, predominantly 4,4’-diphenylmethane diisocyanate or 4,4′-dicyclohexylmethane diisocyanate (HMDI) [1,2,5,6,7], while the soft segments (SSs) consist of long-chain diols, such as polyester, polyether and polycarbonate diols [1,2,3,8,9,10,11]. Chain extenders may include aliphatic and/or aromatic units, and their appropriate selection allows obtaining polyurethane materials with specific properties. When using aliphatic–aromatic and aromatic unconventional diols, we generally obtain materials that are harder and have a higher modulus of elasticity compared to polymers made from aliphatic diols. Nevertheless, among the non-conventional chain extenders, aliphatic–aromatic diols provide polyurethanes with slightly lower hardness but higher elongation at break [1,12,13]. Both types of such extenders are also used to synthesize polyurethanes with increased thermal stability [14,15] or liquid crystalline properties [16]. Additionally, the introduction of sulfur atoms into the structure of chain extenders improves the optical and adhesive properties as well as the refractive index [17,18], while the introduction of a bromine atom improves the fire resistance [19] of the obtained materials. As is well known, TPUs with polyether SSs have better low-temperature properties and hydrolysis resistance compared to TPUs with polyester SSs. The disadvantage is that they are susceptible to oxidation, including oxidation of metal ions, auto-oxidation and cracking under the influence of environmental factors [20,21,22,23,24]. This results in a deterioration of their mechanical properties and damage to polymeric materials. Significantly improved in vivo and in vitro oxidation resistance [20] and hydrolytic stability [25,26,27,28,29] are demonstrated by TPUs with polycarbonate—type SS. Due to the combination of excellent biostability and biocompatibility of these polymers, as well as high tensile strength and elastic modulus [11,20], they are preferred as biomaterials for long-term implantation. Polycarbonate-based TPUs are used in cardiovascular implants, reconstructive surgery, tissue engineering scaffolds and other specialized biomedical applications [6,11,28,29,30,31]. Similarly to the materials described above, TPUs based on a poly(ε-caprolactone) diol SSs have recently also found application as materials used in medicine, among others, thanks to their good biocompatibility. In turn, their biodegradable properties determine their use in the production of materials with a short implantation time [32,33,34].

The aim of this paper was to study the effect of using (polyether, polyester and polycarbonate) soft segments on the physicochemical properties, structure and crystallinity and thermal as well as antibacterial activity of new types of TPUs. These polymers were synthesized from poly(oxytetramethylene) (PTMO) diols of ${\bar{M}}_{n}$ = 1000 Da and ${\bar{M}}_{n}$ = 2000 Da, a poly(ε-caprolactone) (PCL) diol of ${\bar{M}}_{n}$ = 2000 Da and polycarbonate (PCD) diols of ${\bar{M}}_{n}$ = 1000Da and ${\bar{M}}_{n}$ = 2000 Da as soft segments (SSs), as well as HMDI and the unconventional chain extender [methylenedi(4,1-phenylene)]dimethanol (DMD).

The influence of accelerated aging on the structural, mechanical and thermomechanical properties of TPUs will be presented and discussed in the next publication.

## 2. Materials and Methods

### 2.1. Materials

DMD (*T*_m_ = 119 °C) was obtained at the Department of Polymer Chemistry, Maria Curie-Skłodowska University [35]. PTMOs and PCL were purchased from Aldrich (St. Louis, MO, USA). HMDI (*Desmodur W*^®^) and PCDs (*Desmphen^®^ C2100* and *Desmphen^®^ C2200*) were kindly supplied by Covestro (Leverkusen, Germany). Before its use, the SSs were heated at 90 °C in vacuo for 10 h. Dibutyltin dilaurate (DBTDL) from Merck-Schuchardt (Hohenbrunn, Germany), 1,1,2,2-tetrachloroethane (TChE) and diiodomethane (Aldrich, St. Louis, MO, USA) and redistilled water (Millipore, UMCS, Lublin, Poland) were used as received.

### 2.2. Polymer Synthesis

**Figure 1:**
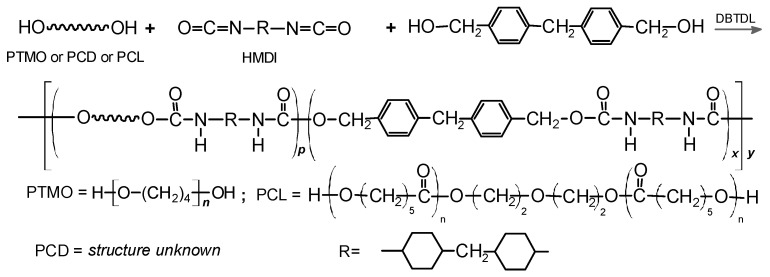
General scheme of synthesis of the TPUs.

TPUs with hard-segment contents of 50 wt% were synthesized by a one-step melt polyaddition from DMD, HMDI and PTMO, PCD or PCL at the NCO/OH molar ratio of 1.05. Firstly, HMDI (0.0105 mol) and oligomer diol and DMD (0.01 mol together) were weighed into a 100 mL reaction flask, and then the flask was placed in an oil bath and heated at 90 °C until the reagents were completely melted, and then DBTDL was added. The contents of the flask were heated, and the temperature was gradually raised to 140 °C, and then the temperature was maintained for 2 h. Figure 1 shows the TPU synthesis scheme, while Table 1 gives the designations and composition of the reagents used to prepare TPU.

### 2.3. Measurements Methods

Reduced viscosities (*η*_red_, dL/g) of 0.5% polymer solution in TChE were measured in an Ubbelohde viscometer (Gliwice, Poland) at 25 °C.

Determination of normal density was performed with the immersion method from ISO 1183-1A [36].

The MFR (melt flow rate) was detemined by using MeltFlow TQ6841 load plastometer manufactured by Ceast (Turin, Italy) and in accordance with ISO 1133 [37].

The ultraviolet–visible (UV/vis) spectra of the polymer compression-molded sheets were taken in the range of 200–900 nm and at a scanning rate of 200 nm/min and by using UV-2550 (Shimadzu, Kioto, Japan) UV spectrophotometer.

The infrared spectra (ATR/FTIR) of the tested polymer compression-molded 1 mm thick sheet were recorded using a Bruker TENSOR 27 spectrophotometer (Ettlingen, Germany), equipped with the PIKE measuring cell (diamond crystal) in the range of 4000–600 cm^−1^. All measurements were recorded at a resolution of 2 cm^−1^ and with 64 scans per sample.

Differential scanning calorimetry (DSC) analysis was made using a Netzsch 204 F1 Phoenix calorimeter (Günzbung, Germany), in accordance with standard ISO 11357-1:2016 [38] and heating–cooling–heating scans. The first heating cycle was from −100 °C to 150 °C, and next the cooling cycle was from 150 °C to −100 °C, and then the second heating scan was from −100 °C to 150 °C. The samples of 10.0 ± 0.5 mg were measured in aluminum crucibles with a pierced lid, and an empty crucible was used as a reference (a mass of 40 ± 1 mg). The scans were performed at a heating/cooling rate of 10 °C/min under argon atmosphere (gas flow = 30 cm^3^/min). The described transitions were taken from first and second heating scans. Glass-transition temperatures (T_g_s) for the polymer samples were taken as the inflection point on the curves of the heat-capacity changes. Melting temperatures (*T*_m_s) were read at endothermic-peak maxima.

The XRD patterns of the TPU samples were collected using an Empyrean Panalytical X-ray apparatus (Malvern, UK). The data collection was recorded in the range of 8–80° with a step of 0.04°. The obtained patterns were analyzed by applying the WAXSFIT computer program [39]. The program resolves a diffraction curve on diffraction peaks and amorphous halo, which allows the degree of crystallinity to be estimated.

TGA was carried out using a Netzsch STA 449 F1 Jupiter thermal analyzer (Selb, Germany) at temperatures from 30 to 800 °C under a helium atmosphere (gas flow rate of 20 cm^3^/min) with a heating rate of 10 °C/min. Sample masses of about 10 mg were tested in open aluminum oxide crucibles, and as a reference an empty crucible was employed.

Contact angles (CAs) of TPUs were measured at 20 °C with a contact angle goniometer (KRÜSS GmbH, Hamburg, Germany) with water and diiodomethane droplets. The volume of droplets was 2 μL. Each sample was analyzed five times, and the average value of the contact angle was designated. For calculation of the surface free energy according to the method of Owens, Wendt, Rabel and Kaelble [7], the Krüss ADVANCE (KRÜSS GmbH, Hamburg, Germany) software ver. 1.14.1.16701 was used to determine the sessile drop orientation, and the ellipse fitting method was used for the data analysis.

The formation of a bacterial biofilm on the surface of biomaterials was tested using four bacterial strains: Gram-positive: *S. aureus* (ATCC 25923) and *S. epidermidis* (ATCC 12228); Gram-negative: *E. coli* (ATCC 25922) and *P. aeruginosa* (ATCC 27859). Briefly, 1 × 1.5 cm rectangular-shaped biomaterials (1–2 mm in thickness) were placed in a 24-well plate and sterilized with ethylene oxide. Then, the bacterial suspension at a concentration of 0.5 McFarland standard density (1 × 10^8^ CFU/mL, prepared in Mueller–Hinton broth, Oxoid, Hampshire, UK) was added to the biomaterials. After 24 h of incubation, the percentage of biofilm formation on the surface of the biomaterials was assessed using crystal violet (CV). First, the bacteria around the biomaterials were removed, and then the samples were rinsed with water three times, and a 0.1% solution of crystal violet was added. After 15 min of incubation (37 °C) at room temperature, the CV was removed, the samples were rinsed 3 times with water and 30% acetic acid was added to dissolve the absorbed CV. The absorbance at 550 nm was measured, and the results are presented as a percentage of the control (results for cells seeded in a plate without biomaterials).

The cytotoxicity of biomaterials was assessed according to ISO 10993-5:2009 standard [40] and ISO 10993-12:2012 standard [41]. For this purpose, green monkey kidney cells (GMK, BIOMED Serum and Vaccine Production Plant, Lublin, Poland) were used. The cells were maintained as described in detail previously [42]. Liquid extracts from biomaterials were prepared in cell culture medium—Eagle’s Minimum Essential Medium (EMEM, ATCC, UK). Cell viability after 24 and 48 h of incubation with the extracts was assessed using the MTT assay.

## 3. Results and Discussion

### 3.1. Physicochemical Properties

**Figure 2:**
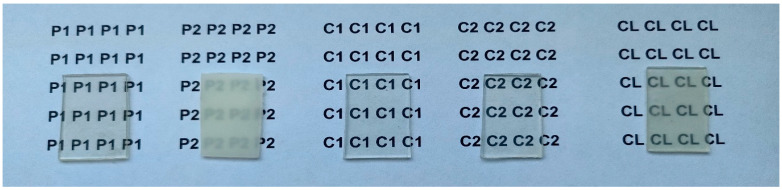
Images of the synthesized polymers.

The polymers obtained were transparent or partially transparent solids (Figure 2) showing high resistance against common organic solvents. All these polymers dissolved at room temperature in TChE and chloroform, but they were insoluble in *N*,*N*-dimethylformamide, *N*,*N*-dimethylacetamide, *N*-methyl-2-pyrrolidone, tetrahydrofuran and dimethyl sulfoxide.

The TPUs obtained were characterized by reduced viscosities (given in Table 2) in the range of 1.40–5.47 dL/g. This indicates their large molar masses, by which the TPUs obtained were characterized by relatively high resistance to organic solvents. Comparing the effect of SS type on viscosity values, it can be seen that the values were higher for polymers obtained from PCL and PCDs. On this basis, it can be concluded that by using SSs of polar nature, it is possible to obtain polymers with higher molar masses than analogous TPUs obtained from less polar SSs.

The polymers obtained showed relatively good transparency, which depended on the type of SS used (Table 2). TPUs obtained using self-crystallizing SSs (P2 and CL) showed lower transparency values than polymers based on other types of SSs. Similar relationships were observed for the determined MFR values. These results suggest that the transparency and MFR of the material depends on its degree of crystallization. This assumption will be explained in further parts of this article when discussing the results of the DSC and XRD analysis.

### 3.2. ATR/FTIR

**Figure 3:**
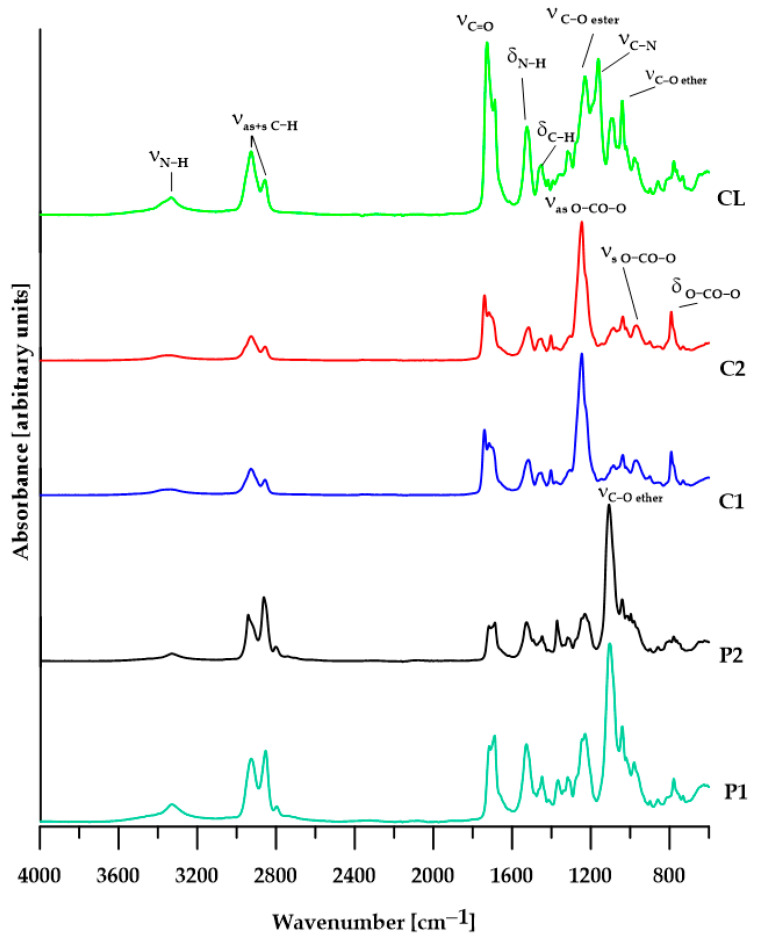
ATR/FTIR spectra of the TPUs.

The chemical structures of the TPUs were confirmed by ATR/FTIR spectroscopy. All of the spectra revealed significant absorptions of the urethane group as well as methylene group but did not show absorption of the isocyanate group at about 2270 cm^−1^, which points to full NCO conversion. The main absorption bands are given below, whereas spectra are shown in Figure 3.

ATR/FTIR for the TPUs from PTMOs (cm^−1^): 3328 (N–H stretching) and 1527 (N–H bending) of the urethane group; 1716–1717 and 1689–1687 (nonbonded and H-bonded C=O stretching of the urethane group); 1107–1103 (C–O stretching of the ether group); 2941–2926 and 2862–2851 (asymmetric and symmetric C–H stretching of CH_2_); 1447 (C–H bending of the cyclohexane ring); 1371–1367 (C–H bending of the aliphatic chain).

ATR/FTIR for the TPUs from PCDs (cm^−1^): 3340–3337 (N–H stretching) and 1517–1515 (N–H bending) of the urethane group; 1741 (nonbonded C=O stretching of the carbonate group); 1717 (nonbonded and H-bonded C=O stretching of the urethane and carbonate group, respectively); 1702 (H-bonded C=O stretching of the urethane group); 1246 and 969 (asymmetric and symmetric O–C–O stretching of carbonate, respectively); 792 (nonplanar bending vibrations of the O–CO–O group.); 2927–2926 and 2856 (asymmetric and symmetric C–H stretching of CH_2_); 1452 (C–H bending of the cyclohexane ring); 1378–1377 (C–H bending of the aliphatic chain).

ATR/FTIR for the TPUs from PCL (cm^−1^): 3333 (N–H stretching) and 1525 (N–H bending) of the urethane group; 1730 (nonbonded C=O stretching of the ester group from PCL); 1687 (H–bonded C=O stretching of the urethane group); 1229 and 978 (asymmetric and symmetric C–O stretching of ester group of PCL, respectively); 778 (O–C–O bending of ester group of PCL); 1064 and 1040 (asymmetric and symmetric C–O–C stretching of ether group from PCL); 2926 and 2856 (asymmetric and symmetric C–H stretching of CH_2_); 1451 (C–H bending of the cyclohexane ring); 1392 (C–H bending of aliphatic chain).

### 3.3. DSC

**Figure 4:**
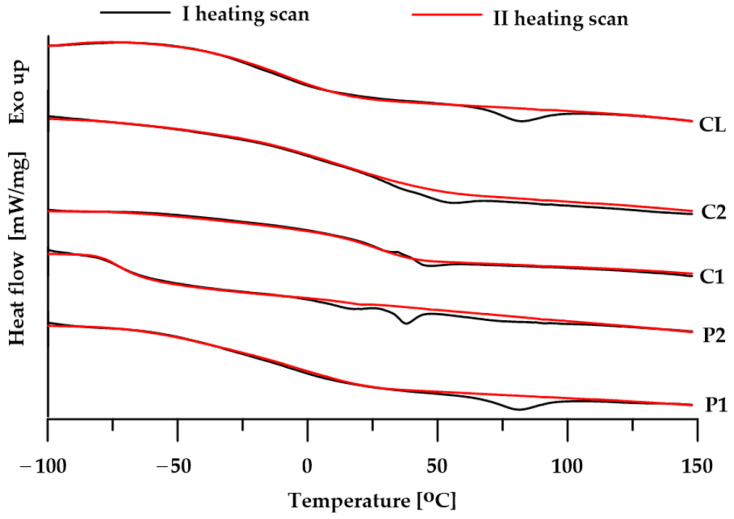
DSC curves of the TPUs.

The DSC analysis of the obtained polymers allowed to determine the physical changes occurring during heating. The obtained DSC curves (Figure 4) from the first heating cycle reveal, among others, glass transitions, from which glass transition temperatures are determined. The glass transition temperatures (Table 3) depend on the type of soft segment used. The polymer obtained from PTMO 2000 has the lowest *T*_g_ value, which indicates that it has the highest degree of microphase separation. The remaining materials were characterized by much smaller differences in *T*_g_ values between pure SSs and the actual polymer, which indicates better phase miscibility and therefore their greater structural homogeneity. Moreover, in the DSC curves from both heating cycles, only the glass transitions of the soft segments are visible, and the glass transitions of the hard segments are not visible.

Apart from the glass transitions, DSC curves from the first heating reveal small endothermic peaks, suggesting the crystalline structure of the obtained materials. In the case of polymers P1 and CL, these peaks may be related to the melting of the less ordered hard segments, while in the case of polymers P2 and C2, these peaks may be related to the melting of the soft segments. DSC curves from the second heating cycle revealed only glassy transformations, which indicates that the obtained materials have a low tendency to crystallize relatively quickly (no crystallization occurred during cooling).

### 3.4. XRD

**Figure 5:**
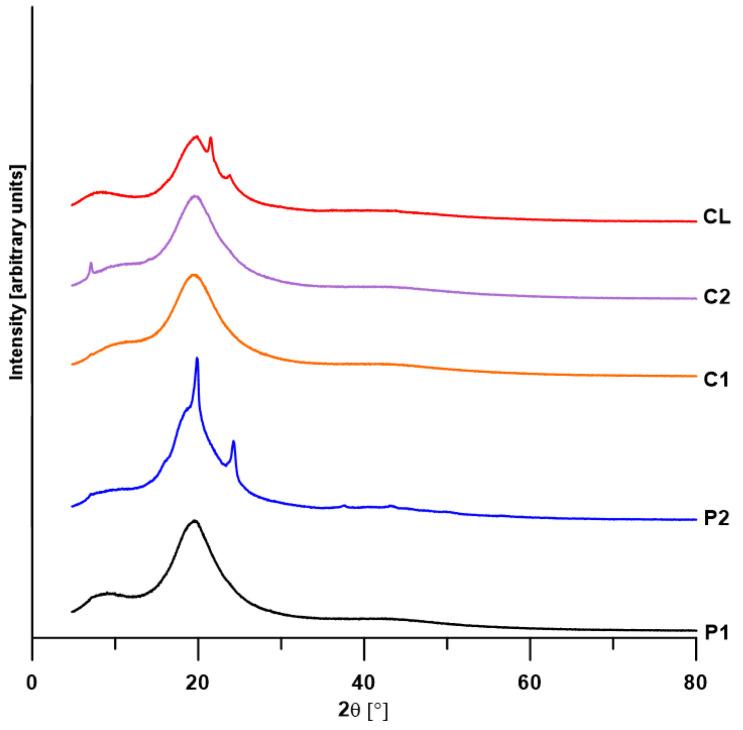
XRD patterns of the TPUs.

**Figure 6:**
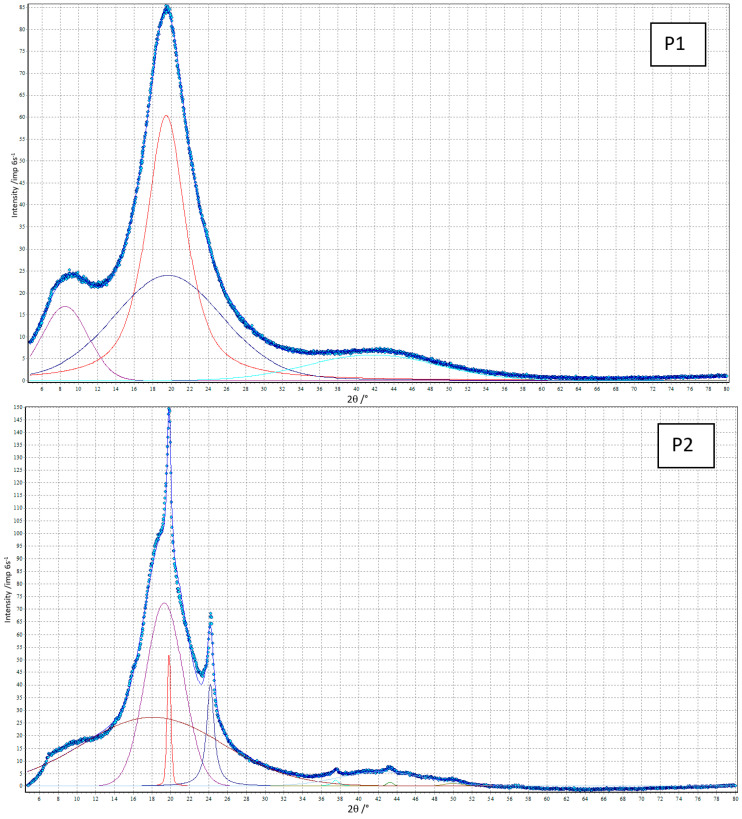

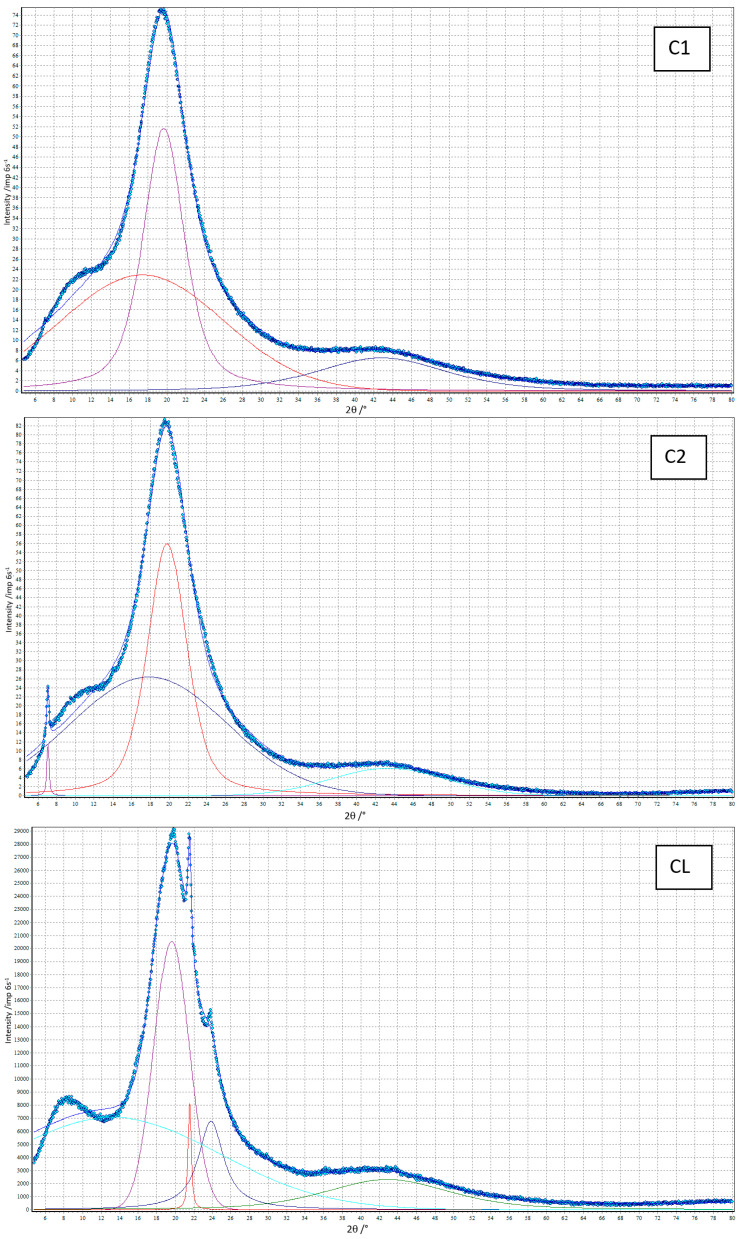
XRD curves (points) of the TPUs resolved into crystalline and amorphous peaks (solid lines).

XRD analysis was performed for all polymers obtained, and the obtained diffractograms are shown in Figure 5. The distributions of the diffractograms obtained using the WAXFIT program confirmed that only some materials showed a partially crystalline structure. Peaks related to the presence of the crystalline phase (FWHM in the range of 0.29–0.97°) were obtained for polymers P2, C2 and CL (Figure 6 and Table 4).

The calculated degrees of crystallinity for these polymers, which are the ratios of the sum of the areas of the crystalline peaks to the sum of the areas of all peaks, were 16.1% for P2, 0.5% for C2 and 1.5% for CL, respectively. The low values of the degrees of crystallinity of the obtained materials confirm the previously obtained results of MFR and transparency (the lower the MFR and transparency, the greater the degree of crystallinity), as well as DSC analysis (the highest degree of microphase separation, and at the same time the highest crystallinity was revealed by the P2 polymer).

### 3.5. Thermal Stability

#### 3.5.1. TGA

**Figure 7:**
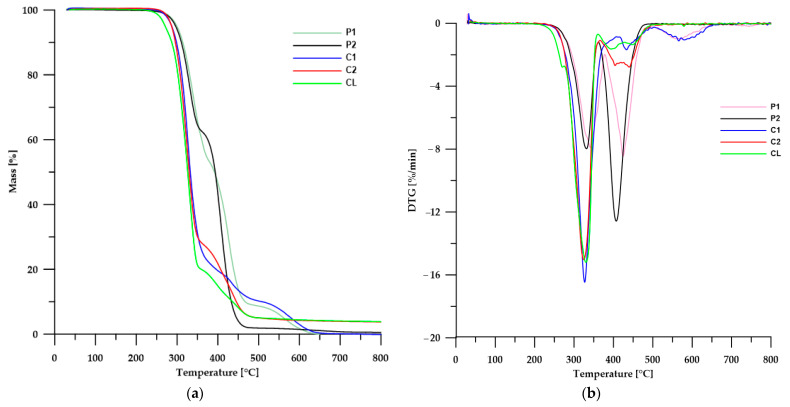
TG (**a**) and DTG (**b**) curves of the TPUs.

The thermal stability of the obtained materials was determined using the thermogravimetric method, and the obtained TG and DTG curves are presented in Figure 7. The numerical data are presented in Table 5.

Based on the data obtained, it can be seen that stability depends on the type of SS used. The polymers obtained from the polyether soft segment were the most stable, which results from the relatively high thermal stability of ether bonds. The least stable polymer was CL, which contains both ether and ester bonds in its structure. Differences in the molar masses of individual SSs did not affect the thermal stability of the polymers.

The DTG curves showed several peaks with different intensities, suggesting a stepwise decomposition of the tested materials. In the case of polymers with a polyether soft segment, decomposition takes place in three (for polymer P1) or two stages (for polymer P2). Peaks with maxima of 341 °C (for P1 and corresponding to a loss of ~47% of mass) and 332 °C (for P2 and corresponding to a loss of ~38% of mass) can be attributed to the decomposition of urethane bonds. The next peaks with maxima of 407 °C (for P2 and corresponding to a loss of ~61% of mass) and 424 °C (for P1 and corresponding to a loss of ~45% of mass) can be connected to the degradation of ether bonds and the degradation of aliphatic chains and aromatic fragments. The peak with a maximum of 571 °C (for P1 and corresponding to a loss of ~9% of mass) may be responsible for the oxidation of solid residues from earlier stages of decomposition. In the case of polymers with a polycarbonate soft segment, the DTG curves reveal three peaks, which indicates a three-stage decomposition. Peaks with maxima at 325 °C (for C2 and with a loss of approximately 73%) and at 327 °C (for C1 and with a loss of approximately 79%) can be attributed to the decomposition of urethane and carbonate bonds. The peaks at a maximum of 433 °C (for C1 with a mass loss of about 11%) and two poorly separated peaks with maxima 404 °C and 442 °C (for C2 and a total mass loss of about 23%) may (as in the case of polymers with a polyether soft segment) be responsible for the decomposition of the aliphatic chains and aromatic chain extender fragments. In turn, the peak with a maximum of about 586 °C (for C1) with a mass loss of about 10% can be attributed to the oxidation of solid residues remaining in the previous stages of decomposition. In the case of the CL polymer, we can distinguish four peaks on the DTG curve with poorly or clearly visible maxima. The first weakly visible peak with a maximum around 271 °C, visible as a shoulder, may be responsible for the decomposition of the ester bond from PCL, while the next peak with a maximum around 330 °C can be attributed to the decomposition of urethane bonds. The next two poorly separated peaks with maxima of 394 °C and 448 °C can be attributed to the distribution of aliphatic and aromatic fragments.

Analyzing the peaks on all DTG curves, it can also be noticed that only in the case of polymers obtained from SSs with molar masses of 1000 Da are there peaks at approximately 586–596 °C, which are responsible for the distributions of solid products formed at lower temperatures. This may be due to the higher content of HS in the polymer compared to analogous polymers obtained from SSs with molar masses of 2000 Da.

The above analysis is consistent with the available literature, which examined in detail the stability of TPUs with various soft segments and unconventional diphenylmethane derivative chain extenders [9,17,18,27,43,44].

#### 3.5.2. TGA-FTIR

**Figure 8:**
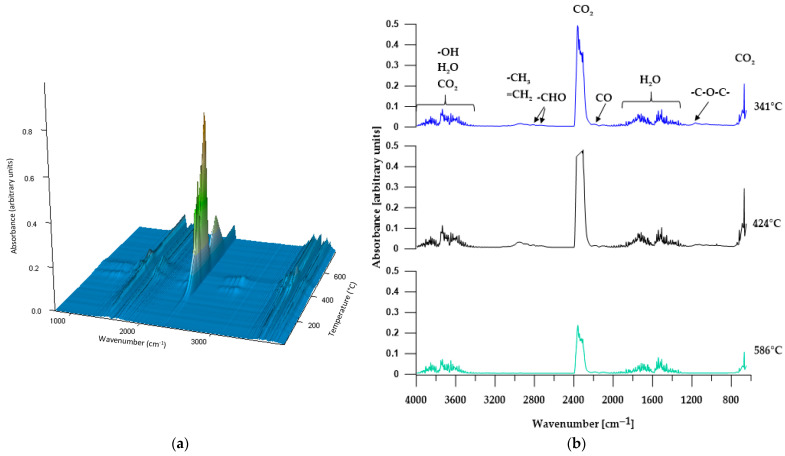
Three-dimensional plot (**a**) and FTIR spectra (**b**) of volatile products obtained during the thermal decomposition of P1.

**Figure 9:**
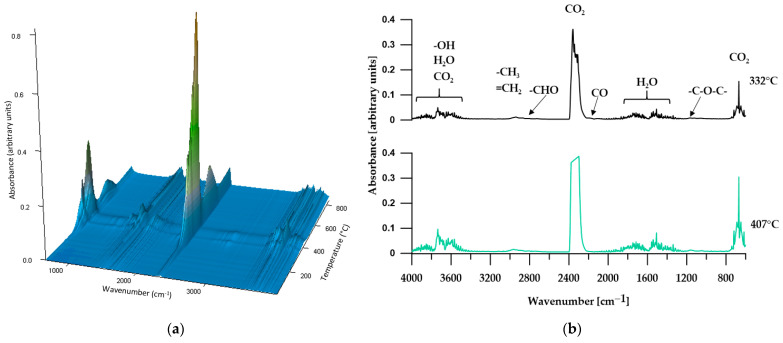
Three-dimensional plot (**a**) and FTIR spectra (**b**) of volatile products obtained during the thermal decomposition of P2.

**Figure 10:**
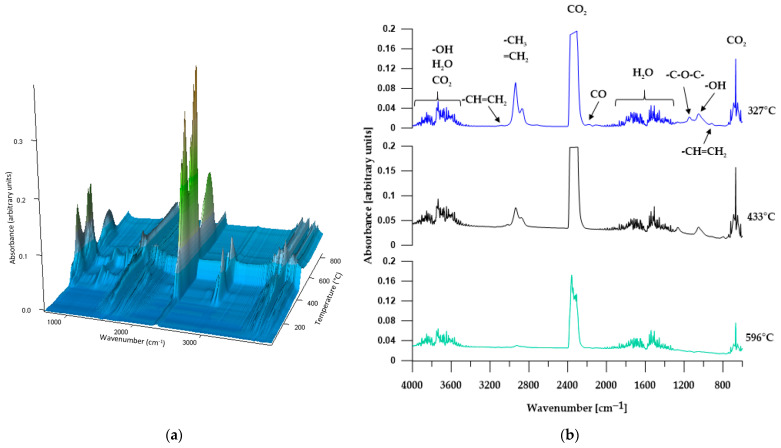
Three-dimensional plot (**a**) and FTIR spectra (**b**) of volatile products obtained during the thermal decomposition of C1.

**Figure 11:**
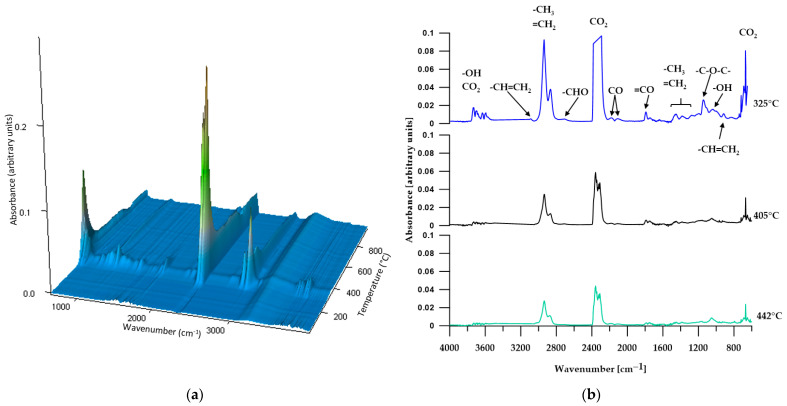
Three-dimensional plot (**a**) and FTIR spectra (**b**) of volatile products obtained during the thermal decomposition of C2.

**Figure 12:**
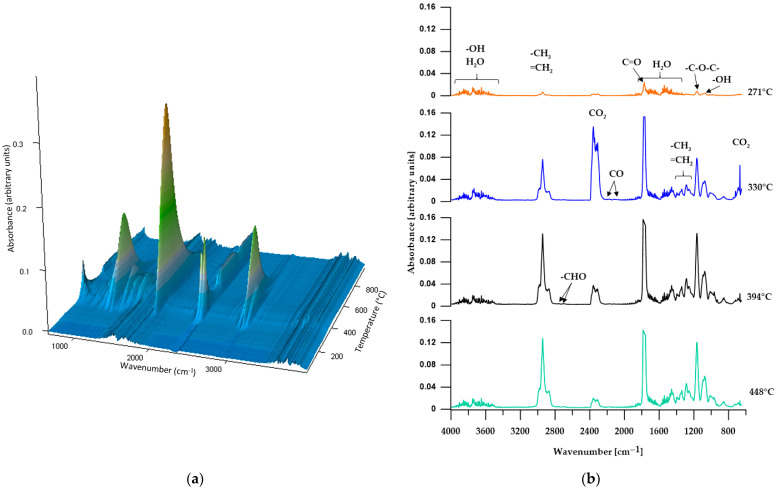
Three-dimensional plot (**a**) and FTIR spectra (**b**) of volatile products obtained during the thermal decomposition of CL.

In order to more comprehensively analyze the distribution of the obtained materials during thermogravimetric analysis, an FTIR analysis of gaseous products released from the samples during heating was performed. Figure 8, Figure 9, Figure 10, Figure 11 and Figure 12 show FTIR spectra of gaseous polymer decomposition products released at temperatures corresponding to the maxima in the DTG curves.

FTIR spectra obtained in all three stages of polymer P1 decomposition (Figure 8b) revealed almost the same absorption bands. These spectra show very strong absorption bands in the range of 2359–2344 cm^−1^ (associated to asymmetric stretching vibrations of C=O) and at 669 cm^−1^ (related to bending vibrations of C=O) and indicating the presence of carbon dioxide in the released gases. These bands have the highest intensity in the second stage of decomposition and the lowest in the third stage, which indicates that the decomposition of the ether bond generates, among other things, carbon dioxide. Moreover, the FTIR spectra from the first and second stages of decomposition show absorption bands in the ranges ~4000–3300 cm^−1^ and 1850–1300 cm^−1^ related to the release of water (stretching and bending vibrations, respectively) and bands at approx. 2190 cm^−1^ (related to C–O stretching vibrations in carbon monoxide), the intensity of which does not change during sample decomposition, however. The presence in the first two stages of the decomposition of small bands at approximately 2954–2946 cm^−1^ and 2869–2863 cm^−1^ (related to symmetric and asymmetric C–H stretching vibrations of methyl and methylene groups) and bands at 1164 cm^−1^ (corresponding to C–O stretching vibrations) and at approx. 2819 and 2716 cm^−1^ (related to the C–H vibrations of the aldehyde group) indicates that, in addition to carbon dioxide and carbon monoxide, the products of thermal decomposition of the P1 polymer are alcohols, ethers and aliphatic aldehydes.

The FTIR spectra of the gaseous decomposition products of polymer P2 (Figure 9b) are the same as those of polymer P1, but in the second stage the absorption band at 2180 cm^−1^, associated with C-O stretching vibrations in carbon monoxide, almost disappears. The difference in the molecular weights of SSs in polymers P1 and P2 does not affect changes in the mechanisms of their thermal decomposition and does not generate additional gaseous decomposition products.

The FTIR spectrum from the first stage of polymer C1 decomposition (Figure 10b, *T*_max_ at 327 °C) shows characteristic absorption bands for carbon dioxide (at 2359–2344 cm^−1^, associated with asymmetric stretching vibrations O=C=O and at 668 cm^−1^, related to degenerate bending vibrations), carbon monoxide (at 2181 and 2114 cm^−1^ corresponding to C=O bending vibrations) and water (~4000–3350 cm^−1^ related to bending vibrations and ~1850–1300 cm^−1^ connected to bending vibrations). Moreover, in the spectrum, one can distinguish bands characteristic of ethers (at approx. 1146 cm^−1^ corresponding to C–O bending vibrations) and alcohols (absorption band at 1051 cm^−1^ associated with C–O bending vibrations). Visible absorption bands at approx. 2938 and 2870 cm^−1^ (characteristic of the asymmetric and symmetric C–H bending vibrations of the methyl and methylene groups) as well as small bands at 3014 cm^−1^ (related to stretching vibrations =C–H) and at 917 cm^−1^ (deformation vibrations of the out-of-plane C–H of the vinyl group) indicate that the released alcohols and ethers are aliphatic and aromatic. The FTIR spectrum from the second stage of decomposition (*T*_max_ at 433 °C) shows almost the same absorption bands as the spectrum from the first stage of decomposition; however, the intensity of the bands characteristic of methyl and methylene groups decreases, and the bands characteristic of carbon monoxide and ethers almost completely disappear. However, the intensity of the bands corresponding to the vibrations of carbon dioxide increases. In turn, the FTIR spectrum of gaseous decomposition products from the third stage of decomposition (*T*_max_ at 596 °C) indicates the presence of water, carbon dioxide and small amounts of aliphatic compounds.

The FTIR spectra from three stages of C2 decomposition (Figure 11b) reveal absorption bands characteristic of carbon dioxide, carbon monoxide, aldehydes and alcohols, with the intensity of the bands decreasing in each subsequent stage of decomposition. Moreover, they release ethers only in the first stage of decomposition, as indicated by the absorption band at 1145 cm^−1^ (C–O stretching vibrations of the ether group). Analyzing the FTIR spectra of the gaseous decomposition products of C1 and C2, it is clearly visible that the type of SS used (or rather, the difference in the molar mass of the SS used) does not affect the type of released compounds.

In the case of the CL polymer, we can distinguish four clear maxima of the mass loss rate, which indicates its four-stage decomposition process. The FTIR spectrum of the gaseous products of decomposition from the first stage (Figure 12b, *T*_max_ at 271 °C) shows absorption bands characteristic of water, carbon dioxide, alcohols and aliphatic ethers, as well as carbonyl compounds. The intensity of the bands is relatively low, which indicates a low rate of sample mass loss at this temperature. FTIR spectra obtained for subsequent stages of decomposition reveal the absorption bands of the above compounds, and their intensities are much higher. Only the intensities of the bands characteristic of water do not change, while the bands for carbon dioxide have the highest intensity in the second stage (*T*_max_ at 330 °C), which decreases in later stages. From the second stage of decomposition, the presence of carbon monoxide can be observed in the gaseous products evolved during decomposition (bands at approx. 2185 and 2115 cm^−1^ associated with stretching vibrations), and bands characteristic of aldehydes are more noticeable (at approximately 2725 cm^−1^).

The use of an unconventional aliphatic–aromatic chain extender did not result in the production of chemical compounds that were too toxic. All polymers decomposed to give low-molecular-weight compounds, among which no amines or diisocyanates were observed, which is important information. Moreover, the lack of heteroatoms in the structure of the chain extender (e.g., sulfur atoms) resulted in the absence among of the gaseous decomposition products of very toxic sulphides, including carbonyl sulphide.

### 3.6. Contact Angles

**Figure 13:**
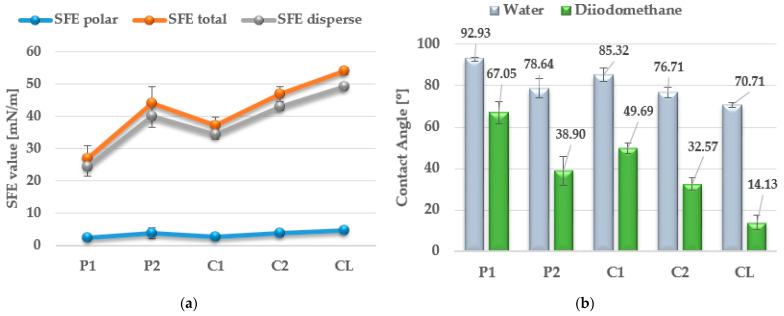
SFE values (**a**) and quantifying the contact angles (**b**) of TPUs.

The surface properties of the obtained polymers were determined from the CA and SFE values, and the obtained results are shown in Figure 13 and Table 6.

In the case of biomaterials, it is important to examine their hydrophobic and hydrophilic properties, which determine their subsequent use. For polyurethane materials used in medicine, SFE values are generally above 50 mN/m [43].

As can be seen from the values presented in Figure 13a, the type of SS used has a significant impact on the SFE values. The lowest SFE value of ca. 25 mN/m was obtained for the P1 polymer, which indicates its high hydrophobicity, while the highest SFE value was recorded for the CL polymer (SFE value of approx. 54 mN/m). Increasing the molar mass of the flexible segment used increased the SFE values, which resulted in a decrease in the surface hydrophobicity of the obtained materials.

As presented in previous work [44], the SFE value can determine the ability of cells to bioadhere on the material surface. Polymers with SFE values above 40 mN/m have good bioadhesive ability; therefore, such materials can be used in orthopedics, where the good ability of the implant to become overgrown with body cells is important.

The CA values shown in Figure 13b confirm the above analysis. The polymer with the most hydrophobic surface was P1 (CA value 92.93°), while the least hydrophobic polymer was CL (CA value 70.71°).

### 3.7. Antimicrobial Activity

#### 3.7.1. Biofilm Formation

**Figure 14:**
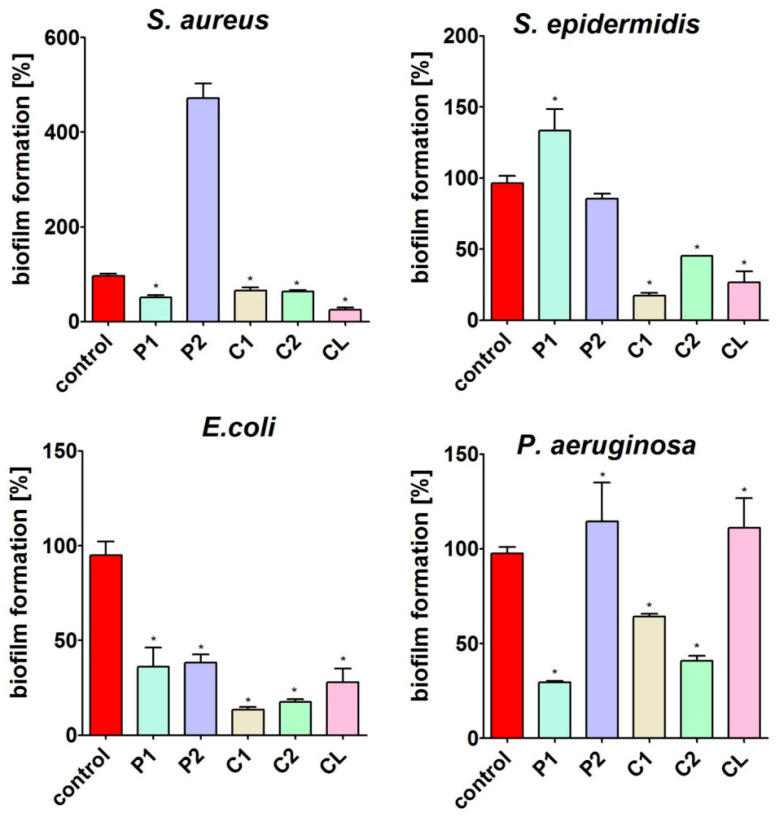
The ability of the surface of materials to prevent the formation of a bacterial biofilm. The percentage of biofilm formation was assessed after 24 h incubation using crystal violet (CV). * Significantly different results compared to control (polystyrene), unpaired Student *t*-test, *p* < 0.05.

The CV test showed that the material surfaces had different levels of ability to inhibit the formation of bacterial biofilm (Figure 14). The surface of the P1 material significantly inhibited the biofilm formation of *S. aureus*, *E. coli* and *P. aeruginosa* but in turn significantly promoted the growth of *S. epidermidis* compared to the control (polystyrene). The surface of the P2 material significantly inhibited biofilm formation only by *E. coli*. Significant inhibition of biofilm formation by *S. aureus*, *S. epidermidis* and *E. coli* was observed for the surface of the CL. Interestingly, the surfaces of the C1 and C2 materials had the ability to significantly decrease the biofilm formation of all tested bacterial strains, indicating that these materials have the most promising antibacterial activity.

As you can see, the chemical nature of the soft segments used significantly affects the antibacterial properties of the tested materials. It seems that this is significantly influenced by the presence of ester groups (mainly carbonate groups) and thus a greater number of hydrogen bonds between the segments. In the case of the CL polymer, the polycaprolactone soft segment contains both an ester bond and an ether bond in its structure, so their antibacterial properties against Gram-negative bacteria are not as favorable as those of polymers with a polycarbonate soft segment.

The obtained results of antimicrobiological tests are very interesting due to the potential use of the obtained materials in medicine. The occurrence of pathogens used for research in the hospital environment and the use of polyurethane materials as implant materials give hope that the polymers obtained may find practical applications after extended research.

#### 3.7.2. Cytotoxicity

**Figure 15:**
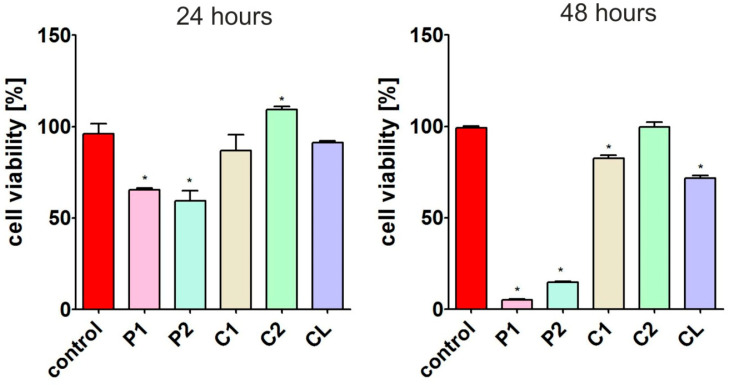
Viability of GMK cells after 24 and 48 h of incubation with extracts obtained from biomaterials. The metabolic activity of the cells was assessed using the MTT assay. * Significantly different results compared to control extract (obtained from polystyrene), unpaired Student *t*-test, *p* < 0.05.

After 24 h of incubation, the MTT assay (Figure 15) showed that extracts from P1 and P2 materials significantly reduced GMK cell viability compared to the control extract. Thus, the metabolic activity of the cells was reduced to approx. 65% (after incubation with the P1 material extract) and to approx. 59% (after incubation with the P2 material extract). After 48 h of incubation of GMK cells with extracts from these materials, an even greater reduction in cell viability was observed. According to the ISO 10993-5:2009 standard [40], if a material extract reduces cell viability below 70%, it should be considered cytotoxic. Thus, the P1 and P2 materials should not be considered promising for biomedical applications. In turn, extracts from C1, C2 and CL did not show the ability to reduce cell viability after 24 h of incubation. However, after 48 h (Figure 15), a slight decrease in the metabolic activity of cells (but statistically significant) to about 82% (after treatment with extract from C1) and 71% (after treatment with extract from CL) was observed. Thus, only the extract from the C2 material did not reduce cell viability after both tested time points compared to the control extract, and it should be considered the most promising for biomedical applications.

The relatively high cytotoxicity of polymers with polyether soft segments indicates that they are more susceptible to hydrolysis than other materials. The low-molecular-weight compounds formed as a result of hydrolysis are toxic, which disqualifies materials with a polyether SS in biomedical applications. In turn, materials with polycarbonate soft segments are most suitable for use in long-term implantation.

## 4. Conclusions

The use of various types of soft segments allowed new polyurethane materials with significantly different properties to be obtained. The obtained materials were characterized by high transparency (up to 87%) and density (1.23 g/cm^3^). The performed ATR/FTIR analysis confirmed the supposed structure of the synthesized materials and in each case confirmed the complete reaction of the isocyanate groups. DSC analysis showed some ordering of the chemical structure of the tested materials, indicating their partially crystalline structure. This was verified by XRD analysis, and based on the obtained results, it was found that the highest degree of crystallinity (16.1%) was demonstrated by the polymer obtained from PTMO 2000 (P2). The other polymers were fully amorphous (P1 and C1) or slightly crystalline (C2 and CL). The obtained polymers were characterized by relatively good thermal stability (up to 248 °C for CL), while the most stable materials were those with a polyether soft segment. The most common gaseous products of polymer decomposition were water, carbon dioxide, carbon monoxide, ethers and aldehydes. In most cases, the surface of the obtained polymers was hydrophilic, except for polymer P1, for which the CA value in water was 92.93°. Increasing the molar masses of the SSs used resulted in a decrease in the CA values and thus an increase in the hydrophilic nature of the obtained materials. Antibacterial activity and cytotoxicity studies showed that polymers obtained from polycarbonate SSs can be effectively used as implantation materials in long-term applications.

Due to the advantageous properties of the materials obtained, further research is planned to expand their current characteristics. The impact of accelerated aging on the thermomechanical and mechanical properties will be analyzed, as well as the hydrolytic stability of the synthesized materials. The results obtained from the planned work will provide an answer as to whether application tests of the manufactured materials will be undertaken.

## Figures and Tables

**Table 1:** Designations and composition used to synthesize the TPUs.

TPU	Soft Segment	Amount of HMDI [mol]	Amount of DMD [mol]	Amount of Soft Segment [mol]
P1	PTMO 1000	0.0105	0.00585	0.00415
P2	PTMO 2000		0.00770	0.00230
C1	*Desmphen C^®^ 2100*		0.00585	0.00415
C2	*Desmphen C^®^ 2200*		0.00770	0.00230
CL	PCL		0.00770	0.00230

**Table 2:** Physicochemical properties and pressing temperatures of the TPUs.

TPU	*η*_red_ [dL/g]	Density [g/cm^3^]	MFR [g/10 min]	Transmittance [%]		Pressing Temperature [°C]
				400 nm	700 nm	
P1	2.17	1.10	13.93 ± 1.92	71.73	84.59	130
P2	1.40	0.98	2.23 ± 0.31	18.18	70.51	135
C1	5.47	1.14	13.57 ± 1.92	76.31	86.95	130
C2	3.49	1.23	11.48 ± 1.30	67.76	82.73	130
CL	2.77	0.96	9.36 ± 1.21	55.77	69.87	145

**Table 3:** DSC data of the TPUs.

TPU	*T_g_* [°C]		*T_m_* [°C]	Δ*H* [J/g]
	I^st^	II^nd^		
P1	−8	−9	81	3.8
P2	−71	−74	38	1.8
C1	31	30	47	0.9
C2	42	23	57	1.2
CL	−17	−11	83	3.3

I^st^, II^nd^—first and second heating scan, respectively.

**Table 4:** XRD data of the TPUs.

TPU	Degree of Crystallinity [%]	2θ [°]	FWHM [°]	Area of Diffraction Peak [Arbitrary Units]
P1	0	8.52	5.88	25
		19.45	5.12	100
		19.65	13.93	87
		41.74	16.76	24
P2	16.1	18.06	17.77	100
		19.31	4.72	71
		19.81 ^a^	0.48 ^a^	6 ^a^
		24.20 ^a^	0.97 ^a^	12 ^a^
		37.49 ^a^	0.84 ^a^	1 ^a^
		42.40	10.61	13
		43.27 ^a^	0.80 ^a^	1 ^a^
		49.96	2.29	1
C1	0	17.39	20.34	100
		19.66	5.21	72
		42.79	16.26	26
C2	0.5	7.08 ^a^	0.29 ^a^	1 ^a^
		17.83	19.66	100
		19.78	5.00	65
		43.35	14.48	17
CL	1.5	13.19	27.83	100
		19.59	4.51	55
		21.54 ^a^	0.43 ^a^	3 ^a^
		23.86	3.32	19
		42.86	15.83	25

^a^ Crystalline peak.

**Table 5:** TGA data of the TPUs.

TPU	*T*_1_ ^1^ [°C]	*T*_5_ ^2^ [°C]	*T*_50_ ^3^ [°C]	*T*_max_ ^4^ [°C]
P1	271	300	392	341; 424; 586
P2	267	297	393	332; 407
C1	266	287	332	327; 433; 596
C2	270	287	330	325; 405; 442
CL	248	271	326	271; 330; 394; 448

^1,2,3^ The temperatures of 1, 5 and 50% mass loss, respectively; ^4^ the temperatures of maximum rate of mass loss.

**Table 6:** Images of sessile drops during CA measurements.

	P1	P2	C1	C2	CL
Water	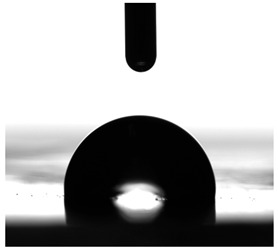	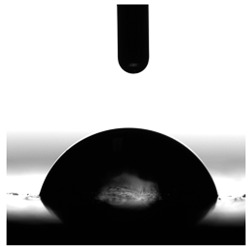	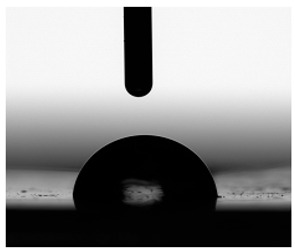	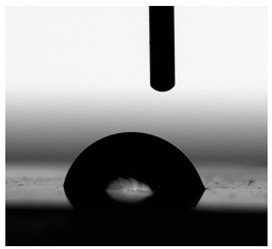	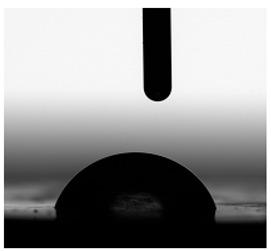
Diiodomethane	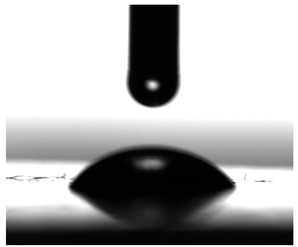	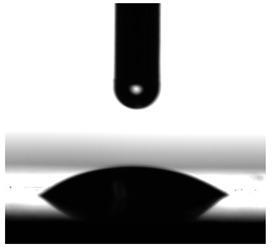	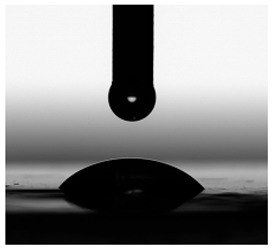	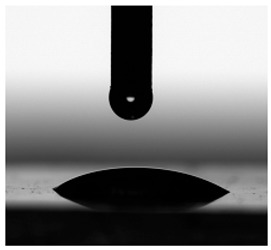	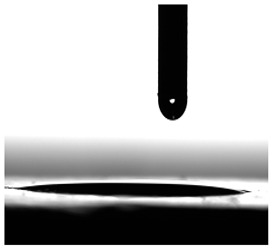

## Data Availability

Data are contained within the article.
